# Novel Personalized Score Predicts Risk for Postoperative Biliary Leak in Liver Surgery—a Retrospective Database Analysis

**DOI:** 10.1007/s11605-022-05366-1

**Published:** 2022-06-17

**Authors:** Carina Riediger, Raphael Hoffmann, Steffen Löck, Esther Giehl-Brown, Sandra Dennler, Christoph Kahlert, Jürgen Weitz

**Affiliations:** 1 Department of Visceral, Thoracic and Vascular Surgery, University Hospital Carl Gustav Carus, Technische Universität Dresden, Dresden, Germany; 2 National Center for Tumor Diseases (NCT/UCC), Dresden, Germany; 3 OncoRay - National Center for Radiation Research in Oncology, Technische Universität Dresden, Helmholtz-Zentrum Dresden-Rossendorf, Dresden, Germany; 4 Faculty of Medicine and University Hospital Carl Gustav Carus, Technische Universität Dresden, Dresden, Germany; 5 Helmholtz-Zentrum Dresden - Rossendorf (HZDR), Dresden, Germany

**Keywords:** Biliary leakage risk score, Hepatectomy, Morbidity, Mortality

## Abstract

**Background:**

The number of liver resections is constantly rising over the last decades. Despite the reduction of overall mortality and morbidity in liver surgery, biliary leakage is still a relevant postoperative complication that can lead to a fatal postoperative course. Aim of this analysis is the identification of specific risk factors for postoperative biliary complications after liver resections and the development of a predictive biliary leakage risk score.

**Methods:**

A single-center, retrospective analysis of 844 liver resections performed in the Department of Visceral, Thoracic and Vascular Surgery, Technische Universität Dresden, between 1/2013 and 12/2019 is conducted to identify risk factors for postoperative biliary leakage and a risk score for biliary leakage after hepatectomy is established based on multivariate regression. The score has been validated by an independent validation cohort consisting of 142 patients.

**Results:**

Overall morbidity is 43.1% with 36% surgical complications and an overall mortality of 4.3%. Biliary leakage occurred in 15.8% of patients. A predictive score for postoperative biliary leakage based on age, major resection, pretreatment with FOLFOX/cetuximab and operating time is created. Patients are stratified to low (< 15%) and high (> 15%) risk with a sensitivity of 67.4% and a specificity of 70.7% in development cohort and a specificity of 68.2% and sensitivity of 75.8% in validation cohort.

**Conclusions:**

The presented score is robust and has been validated in an independent patient cohort. Depending on the calculated risk, prevention or early treatment can be initiated to avoid bile leakage and to improve postoperative course.

**Supplementary Information:**

The online version contains supplementary material available at 10.1007/s11605-022-05366-1.

## Introduction

In recent years, the number of liver resections is rising due to improvements of anesthesiological and surgical techniques as well as widening indications due to more progressive multimodal oncological concepts.^1–4^ There is not only an increase in minimally invasive liver resections as part of the Enhanced Recovery After Surgery (ERAS) concept, but also a rising number of more complex hepatectomies in multimodal oncological concepts (staged hepatectomy, associating liver partition and portal vein ligation for staged hepatectomy (ALPPS)) with biliary and/or vascular reconstruction.^5,6^

Liver surgery is one of the technically most demanding surgical disciplines due to complex anatomy of the blood vessels and bile ducts. In addition, the high intrahepatic blood volume and the central role of the liver in the metabolic system lead to risk for postoperative morbidity and mortality. Since the first liver resection by Professor Langenbuch in 1888, morbidity and mortality were impressively reduced.^1,7,8^ Especially bleeding complications are nowadays a well-controlled aspect in liver surgery. The main complications after liver surgery are postoperative liver failure, infectious complications, and biliary complications.^9^

Many efforts have been made to reduce the incidence of postoperative bile leakage (POBL) by using new transection tools, intraoperative leakage tests as the white test, and the placement of protective tubes as T-tubes or Neuhaus drains.^10–12^

Despite all improvements in liver surgery, postoperative biliary complications are constantly occurring. Incidences of reported POBL vary between 3.6 and 31%.^13–17^ The wide range is depending on the characteristics of the analyzed cohort, the diagnosis, and mode of surgery as well as the underlying definition of bile leakage. In 2010, the definition for biliary leakages of the International Study Group of Liver Surgery (ISGLS) was published: Three grades of bile leakages are discerned: grade A bile leaks require no change in patient’s management and intraoperatively placed drains remain no longer than 7 postoperative days (PODs), grade B leakages require interventional treatment other than surgery, and grade C need surgical revision.^18^ The ISGLS definition for bile leakages is one of the most used definitions for biliary leakage in liver surgery. According to the ISGLS definition, most studies report bile leakages between 10 and 15%. Postoperative biliary complications can lead to a fatal postoperative course with increased morbidity and increased severity of complications (> Clavien II), prolonged hospital stay, and higher postoperative mortality.^19^

Aim of this study was the analysis of risk factors for POBL and the establishment and validation of a predictive risk score for POBL after liver surgery.

## Materials and Methods

### Patients

All patients who received liver resection in the in the Department of Visceral, Thoracic and Vascular Surgery in the University Hospital Dresden between 01/2013 and 12/2020 were prospectively collected in an electronic database.

### Main Development Cohort

Only patients with malignant diagnoses who underwent resection between 01/2013 and 12/2019 were included in further data analysis for the development of the risk score. Patients receiving simultaneous major visceral resection (e.g., gastrectomy, pancreatectomy) were excluded from analysis.

### Validation Cohort

Patients with malignant diagnoses who underwent surgery between 01/2020 and 10/2020 were excluded in the initial data analysis, but were used as an independent cohort for validation of the newly established biliary leakage risk score.

### Database

Database contained 155 parameters that were recorded for each patient including patient’s characteristics (with diagnoses, comorbidities, risk factors such as alcohol and medication) as well as oncological/interventional pretreatments, intraoperative parameters (e.g., transection technique, blood transfusion, pedicle or cava clamping) and postoperative parameters (such as morbidity, mortality, reoperation, hospital, and ICU stay). Database was regularly revised, corrected, and updated.

### Surgery

Liver surgery is highly standardized in our center. Open as well as minimally invasive or hybrid approaches are performed. Intraoperative ultrasound is standard of care.

If needed, clamping of the vena cava and/or Pringles pedicle clamping are performed for reduction of intraoperative blood loss.

Transection of liver parenchyma is mainly performed by Ligasure®, Crush Clamp technique or Stapler (EndoGIA). If possible, major resections are performed by using the pedicle or Glisson’s approach and simultaneous cholecystectomy and the so-called “white test” with lipofundin® is used for the detection of bile leakages.

Minor liver resections contain all atypical resection of either number as well as anatomical resections of up to three anatomical segments. Major resections are defined as all anatomical resections of more than three anatomical segments. Biliodigestive anastomoses are performed as end-to-side hepaticojejunostmy with (mostly transmesocolic) Roux-y anastomosis using PDS 5/0 single stitches. Neuhaus drains are only used in high-risk situations. Abdominal laminar flow drains are routinely used for major resections and in selected minor resections.

### Bile Leakage Definition

In this analysis, any kind of biliary complication is classified as “biliary leakage” (including bilioma and insufficiency of biliodigestive anastomosis) according to ISGLS.^18^

### Study Design

The selected cohort was analyzed for patient’s characteristics, diagnosis, pretreatments, surgical procedures, and postoperative outcome including morbidity with detailed analysis of complications, biliary leakage, 30-day mortality, and reoperation rate.

According to occurrence of POBL, patients were divided into two groups and comparative analysis was performed to identify risk factors for POBL and to analyze the impact of POBL on the postoperative course.

Univariate analysis of the development cohort for the occurrence of POBL was performed and significant parameters were included in a forward selection logistic regression model based on the likelihood ratio with *p*-values for inclusion of 0.05 and exclusion of 0.1 to identify significant predictive risk factors.

### Biliary Leakage Risk Score (BLRS)

Based on the multivariate logistic regression model, a biliary risk score was calculated for every patient as the probability for biliary leakage given by the logistic function (based on the main cohort resected between 2013 and 2019). The area under the receiver operating characteristics curve (AUC) was calculated with asymptotic 95% confidence intervals. Two approaches for patient stratification were then tested. One cutoff was defined based on the Youden index to stratify patients into a group of low and high risk for POBL. Sensitivity and specificity were evaluated.

The biliary risk score was than validated by using the group of patients excluded from the cohort analyzed for biliary leakages (patients receiving liver resection between 1/2020 and 10/2020). For this purpose, the developed logistic regression model and the cutoffs were applied unmodified to the validation cohort. The AUC, sensitivity, and specificity as well as differences in the actual risk of POBL were assessed.

### Statistical Analysis

Statistical analysis was performed using IBM SPSS Statistics software, version 27 (SPSS Inc., Chicago, IL, USA). Data are described by means ± standard deviation or, where appropriate, by median values and interquartile range [IQR]. Explorative group comparisons were conducted using the Mann–Whitney *U* test for continuous variables and the chi-squared test for categorical variables. All statistical tests were conducted two-sided, and a *p*-value < 0.05 was considered statistically significant. In order to sustain maximum statistical power, no correction of *p*-values in the course of multiple testing was performed. However, results of all formal comparisons are thoroughly reported so that an informal adjustment of *p*-values may be performed.

### Ethical Aspects

The study was conducted according to the Declaration of Helsinki with waivers of informed consent of all patients. Ethical approval by local ethics committee was obtained before analysis (Number: BO-EK-540122020).

## Results

### Patient’s Characteristics and Surgical Procedures

Between 01/2013 and 12/2019, *n* = 1034 liver resections in *n* = 862 patients were performed in the University Hospital Dresden, Technische Universität Dresden.

According to inclusion and exclusion criteria, *n* = 844 resections were included in the analysis. 65.5% of patients were male and 34.5% female with a median age of 65 years (mean 63.5; + / − STD 11.9 years). 46.4% of all liver resections were classified as major resections, defined as resection of more than three anatomical segments including ALPPS in 5.3% of all procedures. 51.5% were classified as minor resections including 30.7% singular or multiple non-anatomical resections and 20.9% minor anatomical resections. Most liver resections were performed by open approach (91.5%), while 8.5% were performed minimally invasive (with a rising number over the last years).

Detailed information of patient’s characteristics is displayed in Table 1.

**Table 1:** Patient’s characteristics and surgical procedures

		Whole cohort *n* = 844		Group 1 (without POBL) *n* = 711		Group 2 (with POBL) *n* = 133		*p*-value
		*n*	%	*n*	%	*n*	%	
Gender								
Male		553	65.5%	463	65.1%	90	67.7%	0.570
Female		291	34.5%	248	34.9%	43	32.3%	
Age [years]		63.5 ± 11.9		63.0 ± 12.0		66.1 ± 11.1		0.006
Body mass index [kg/m^2^]		26.8 ± 5.5		26.8 ± 5.4		26.9 ± 5.7		0.778
ASA Classification	ASA I	13	1.5%	13	1.8%	0	0%	0362
	ASA II	291	34.5%	246	34.6%	45	33.8%	
	ASA III	535	63.4%	447	62.9%	88	66.2%	
	ASA IV	3	0.4%	3	0.4%	0	0%	
Diabetes mellitus		224	26.5%	185	26.0%	39	29.3%	0.428
Renal insufficiency		115	13.6%	95	13.4%	20	15.0%	0.605
Smoking		116	13.7%	100	14.1%	16	12.0%	0.532
Alcohol abuse		46	5.5%	44	6.2%	2	1.5%	0.029
Aspirine intake		101	12.0%	81	11.4%	20	15.0%	0.235
CASH		46	5.5%	35	4.9%	11	8.3%	0.118
Liver fibrosis or cirhosis		429	50.8%	352	49.5%	77	57.9%	0.076
Preceeding liver resection		218	25.8%	187	26.3%	31	23.3%	0.469
Neoadjuvant chemotherapy		154	18.3%	135	19.0%	19	14.3%	0.198
Neoadjuvant antibody treatment		11	1.3%	10	1.4%	1	0.8%	0.541
Neoadjuvante combined chemo- and antibody therapy		187	22.2%	157	22.1%	30	22.6%	0.904
Monotherapy FOLFOX		48	5.7%	39	5.5%	9	6.8%	0.558
Monotherapy FOLFIRI		12	1.4%	12	1.7%	0	0%	0.131
Monotherapy 5-FU		8	1.0%	6	0.8%	2	1.5%	0.471
Monotherapy FOLFOXIRI		33	3.9%	32	4.5%	1	0.8%	0.041
Combined treatment FOLFOX und cetuximab		25	3.0%	17	2.4%	8	6.0%	0.024
Combined treatment FOLFIRI und cetuximab		33	3.9%	28	3.9%	5	3.8%	0.922
Combined treatment FOLFIRI und Bevacizumab		14	1.7%	12	1.7%	2	1.5%	0.879
Preoperative interventions		160	19.0%	124	17.4%	36	27.1%	0.009
PVE TACE ERCP + Stent/ PTCD		115	13.6%	93	13.1%	22	16.5%	0.003
		18	2.1%	13	1.8%	5	3.8%	
		19	2.3%	11	1.6%	8	6.0%	
Diagnosis								
Colorectal liver metastases		417	49.4%	358	50.4%	59	44.4%	0.205
Hepatocellular carcinoma		153	18.1%	135	19.0%	18	13.5%	0.134
Cholangiocarcinoma (overall)		175	20.7%	126	17.7%	49	36.8%	< 0.001
Gall bladder carcinoma Intrahepatic cholangiocarcinoma Klatskin tumor		23	2.7%	18	2.5%	5	3.8%	0.425
		109	12.9%	84	11.8%	25	18.8%	0.028
		43	5.1%	24	3.4%	19	14.3%	< 0.001
Neuroendocrine liver metastases		32	3.8%	31	4.4%	1	0.8%	0.046
NCNN liver metastases		65	7.7%	60	8.4%	5	3.8%	0.063
Surgical procedures								
Major resection		347	41.1%	262	36.9%	85	63.9%	< 0.001
ALPPS		45	5.3%	36	5.1%	9	6.8%	0.422
Minor resection (overall)		435	51.5%	397	55.8%	38	28.6%	< 0.001
Minor anatomical resection		176	20.9%	159	22.4%	17	12.8%	0.013
Number of segments in anatomical minor resections		2.05 ± 1.99		1.89 ± 1.95		2.94 ± 1.98		< 0.001
Minor non-anatomical resection		259	30.7%	238	33.5%	21	15.8%	< 0.001
Number of segments in non-anatomical minor resections		2.21 ± 1.68		2.24 ± 1.72		1.95 ± 1.07		0.238
Operating time (min)		265.8 ± 133.1		252.4 ± 126.0		337.5 ± 146.7		0.012
Pringle clamping		189	22.4%	156	21.9%	33	24.8%	0.466
VCI clamping		111	13.2%	82	11.5%	29	21.8%	0.001
VCI resection		50	5.9%	36	5.1%	14	10.5%	0.014
Biliodigestive anastomosis		100	11.9%	62	8.7%	38	28.6%	< 0.001
Portal vein reconstruction		22	2.6%	11	1.6%	11	8.3%	< 0.001
Hepatic artery reconstruction		8	1.0%	4	0.6%	4	3.0%	0.008
Lymph node dissection		264	31.3%	213	30.0%	51	38.4%	0.055
Intraoperative transfusion of PRBC		0.78 ± 1.75		0.68 ± 1.63		1.32 ± 2.20		< 0.001
Intraoperative transfusion of platelets		0.04 ± 0.28		0.03 ± 0.23		0.10 ± 0.48		0.049
Intraoperative transfusion of FFP		1.30 ± 2.77		1.14 ± 2.62		2.14 ± 3.36		< 0.001

*ASA* Amercian Association of Anesthesiologists, *CASH* chemotherapy-associated steatohepatitis, *PVE* portal vein embolization, *TACE* transarterial chemoembolization, *ERCP* endoscopic retrograde cholangiopancreatociscopy, *PTCD* percutaneous transhepatic cholangio drain, *NCNN* non-colorectal, non-neuroendocrine, *ALPPS* associating liver partition and portal vein ligation for staged hepatectomy, *VCI* vena cava inferior, *PRBC* packed red blood cells, *FFP* fresh frozen plasma

### Postoperative Outcome

Overall morbidity was 43.1% with 36% related to surgical complications. The most frequent complication was biliary leakage with 15.8% (133/844) followed by wound infections in 13.6% and non-biliary fluid collections in 9%. Non-surgical complications occurred in 23.8% consisting of 13.2% pulmonary complications, 6% renal complications, and 6.9% cardiovascular complications. As defined above, biliary leakage also included biliomas (*n* = 71) and leak of the biliodigestive anastomosis (*n* = 21). According to ISGLS, *n* = 29 were classified grade A, *n* = 78 grade B and *n* = 26 grade C (Table 2).

**Table 2:** Postoperative data

		Whole cohort *n* = 844		Group 1 (without POBL) *n* = 711		Group 2 (with POBL) *n* = 133		*p*-value
		*n*	%	*n*	%	*n*	%	
Surgical revision	Excluding ISGL Grade C leakage	99	11.7%	67	9.4%	32	24.1%	< 0.001
	Including ISGL Grade C leakage	125	14.8%	67%	9.4%	58	43.6%	< 0.001
ICU stay [days]		Median 1 IQR 0.0–4.0		Median 1 IQR 1.0–3.0		Median 4 IQR 2.0–12.75		< 0.001
Hospital stay [days]		Median 13 IQR 9.0–23.0		Median 12 IQR 9.0–12.0		Median 26 IQR 16.25–45.0		< 0.001
30-day mortality		36	4.3%	24	3.4%	12	9.0%	0.003
Postoperative occurrence of septical complications		44	5.2%	25	3.5%	19	14.3%	< 0.001
Complications according to Clavien								
I		54	6.4%	45	6.3%	9	6.8%	< 0.001
II		71	8.4%	56	7.9%	15	11.3%	
III		149	17.7%	81	11.4%	68	51.1%	
IV		40	4.7%	18	2.5%	22	16.5%	
V		50	5.9%	31	4.4%	19	14.3%	
Number of complications								
1		145	17.2%	120	16.9%	25	18.8%	< 0.001
1–3		128	15.2%	80	11.3%	48	36.1%	
> 3		91	10.8%	31	4.4%	60	45.1%	

*ISGL* International Study Group of Liver Surgery, *ICU* intensive care unit

### Comparative Analysis and Univariate Analyses for Postoperative Bile Leakage

#### Patients’ Characteristics, Diagnoses, and Surgical Procedures

Comparative analysis of patients with versus without POBL was performed to identify risk factors and impact of the biliary leakage on the postoperative outcome after liver resection. Group 1 (*n* = 711) developed no bile leakage while group 2 (*n* = 133) showed POBL. In the bile leakage group (group 2), patients were older, received significantly more often perioperative intervention (ERCP/Stenting), and were significantly more common pretreated with FOLFOXIRI or FOLFOX/cetuximab (Table 1).

Regarding diagnoses and surgical procedures in both groups, cholangiocarcinomas were significantly more prevalent in the bile leakage group, especially Klatskin tumors, while liver metastases of neuroendocrine carcinomas were significantly more often resected without biliary leakage (Table 1). In group 2, significantly more patients underwent major resections with 63.9% (*n* = 85) compared to group 1 with 36.8% (*n* = 262) (*p* < 0.001). Percentage of patients receiving ALPPS procedure was not significantly different between group 1 (5.1%) and group 2 (6.8%) (*p* = 0.422). Non-anatomical resections were significantly less often performed in the biliary leak group (15.8%) compared to group 1 (33.5%) (*p* < 0.001). Patients without bile leakage received significantly more laparoscopic resections (9.4%) than patients with postoperative leakage (3.8%) (*p* = 0.032). Operating time was about 90 min longer in patients with postoperative bile leakage with a median operating time of 311 min (IQR 235.5–417.5 min) in group 2 compared to group 1 with 220 min (IQR 160–324) (*p* = 0.012). Patients with bile leak received significantly more often biliodigestive anastomosis and portal vein reconstruction as well as reconstruction of the hepatic artery. Detailed information are given in Table 1.

#### Postoperative Outcome

To evaluate the impact of bile leakages on postoperative outcome, postoperative parameters were compared as well. Patients with bile leakage had significantly higher postoperative 30-day mortality (9% vs. 3.4%, *p* = 0.003) with significantly longer median ICU stay of 4 vs. 1 day (*p* < 0.001) and a significantly prolonged hospital stay (median 26 vs. 12 days, *p* < 0.001). In addition, patients with bile leakage tended to have more than one complication and developed more severe complications according to Clavien-Dindo (Table 2).

### Multivariate Analysis for Postoperative Bile Leakage (POBL) and Biliary Leakage Risk Score (BLRS)

Multivariate analysis of all pre- and intraoperative parameters that were significantly associated with POBL in univariate analyses was performed. Multivariate analysis with forward selection revealed patients’ age (in years), major resections (yes: 1, no: 0), preoperative treatment with FOLFOX and cetuximab (yes 1, no 0), and operating time (in minutes) as additional significant risk factors (Table 3). Based on the results of our multivariate analysis, the risk for postoperative bile leakage (RPOBL) was calculated as follows:

**Table 3:** Significant parameters for poostoperative biliary leakage after multivariate logistic regression analysis

Parameter	Regression coefficient	Odd’s ratio (95% CI)	*p*-value
Age	0.026	1.026 (1.007–1.047)	0.008
Major resection	0.841	2.320 (1.489–3.614)	< 0.001
Combined treatment FOLFOX und Cetuximab	1.253	3.500 (1.393–8.794)	0.008
Operating time	0.004	1.004 (1.003–1.006)	< 0.001
Constant	− 5.039	0.006	< 0.001

*CI* confidence interval

$$\mathrm{RPOBL }= 0.026 \times \mathrm{ Age }+ 0.841 \times \mathrm{ Major resection }+ 1.253 \times \mathrm{ FOLFOX and cetuximab }+ 0.004 \times \mathrm{ OP time}$$

The numbers included in this formula represents the impact of each factor on the risk of biliary leakage. Pretreatment with FOLFOX and cetuximab has a somewhat larger impact (1.253) than major resection (0.841), while the numbers in front of age (0.026) and operating time (0.004) specify the risk increase per year and minute, respectively. This formula can be translated to an expected biliary leakage probability, using the biliary risk score (BLRS):

$$\mathrm{BLRS }= 1 / (1+\mathrm{exp }(5.039 -\mathrm{ RPOBL})).$$

Both formulas can be easily implemented in common software for tabular calculation and provide a probability estimate for POBL based on the four given parameters (BLRS Calculator—Supplement 1).

**Fig. 1:**
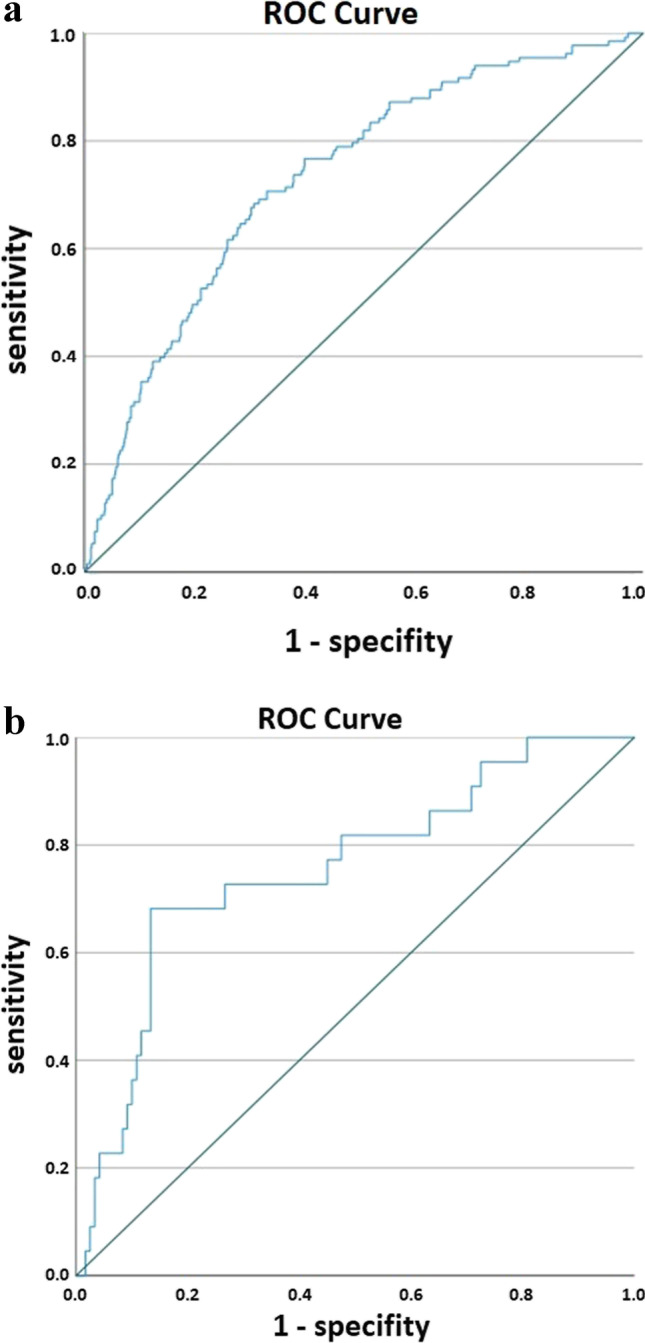
**a** Receiver operating characteristics (ROC) curve for the probability for occurrence of postoperative bile leakage with a specificity of 67.4% and sensitivity of 70.7% based on the development cohort. **b** The ROC curve for the validation cohort. The area under the curve is 0.755 (95CI 0.641–0.870). Given the asymptotic CI > 0.5 demonstrates the positive validation of the BLRS. Based on the calculated Youden index of 0.149 with a sensitivity of 68.2% and a specificity of 75.8%

For the development cohort, the AUC was 0.732 (95% confidence interval 0.687–0.778). The maximum Youden index was reached at a cutoff of 14.9% leading to a specificity of 67.4% and sensitivity of 70.7% for the classification of the patients into a high-risk and low-risk group for POBL (Fig. 1a). A good agreement with the actual observed fractions of POBL was observed, and the groups showed significant differences in the occurrence of bile leakage (Table 4).

**Table 4:** Biliary leakage risk score (BLRS) based on the development cohort (*n* = 844 patients)

BLRS model	BLRS	Risk according to BLRS (in %)	*n* = 844	POBL		Odd’s ratio (95% CI)	*p*-value
				*n*	%		
2-stage (Binary)	< 0.149	< 15%	518	39	7.5%		
	≥ 0.149	> 15%	326	94	28.8%	4.976 (3.319–7.460)	< 0.001

*BLRS* biliary leakage risk score, *POBL* postoperative biliary leakage, *CI* confidence interval

### Validation of the Biliary Risk Score

For validation of the BLRS, we applied the score to 142 patients who consecutively received liver resection for malignant diagnosis in our hospital between 01/2020 and 10/2020. In the validation cohort, 15.5% (*n* = 22/142) patients developed POBL (comparable with development cohort 15.8%).

The calculated risk according to the BLRS for every patient was compared with the occurrence of POBL (Table 5).

**Table 5:** Validation of the biliary leakage risk score applied to the validation chort (*n* = 142)

BLRS model	BLRS	Risk according to BLRS (in %)	*n* = 142	POBL		Odd’s ratio (95% CI)	*p*-value
				*n*	%		
2-stage (Binary)	< 0.149	< 15%	98	7	7.1%		
	≥ 0.149	> 15%	44	15	34.1%	6.724 (2.499–18.091)	< 0.001

*BLRS* biliary leakage risk score, *POBL* postoperative biliary leakage, *CI* confidence interval

In the validation cohort, the AUC was 0.755 (0.641–0.870), i.e., similar to the development cohort, representing a successful validation.

Based on the cutoff 0.149 for the two risk groups, a sensitivity of 68.2% and a specificity of 75.8% were observed, which was similar to the development cohort (Fig. 1b).

## Discussion

Postoperative biliary leakage is a persistent hurdle in liver surgery despite all technical improvements. Indeed, POBL is one of the most common complications after hepatectomy besides liver failure and minor complications such as surgical wound infections.^7,13^

According to the literature, the incidence of POBL varies between 3.6 and 31%.^13–17^ The great variation of incidence is explained by the fact that different definitions of bile leakages are used and that the analyzed cohorts differ between the studies.

Many analyses are performed in well-selected series either excluding resection and reconstruction of the extrahepatic bile duct (exclusion of BDA), Klatskin tumors or ALPPS procedures, or focusing exclusively on patients resected for HCC or CRLM, e.g.,.^16,20–22^

This is of importance as bile leakage rates in patients receiving BDA are generally comparably higher.

In the present study, analysis for all liver resections for any malignancy including ALPPS and BDA and vascular reconstruction is performed. Any kind of biliary collection is defined as biliary leakage and according to the ISGLS definition our data shows an overall biliary leakage rate of 15.8% (Grade A 29 (3.4%), Grade B 78 (9.2%), and Grade C 26 (3.1%)).

### Impact of Biliary Leakage on Postoperative Outcome

POBL is of importance due to its impact on patients’ postoperative course. Our data show that patients with postoperative bile leakage have significantly increased overall mortality of 9% in the bile leakage group (group 2) compared to 3.4% in patients without bile leakage (group 1) and significantly longer ICU and hospital stay. The number of complications and the severity of complications were significantly increased in group 2. Extended hospital stays, increased non-surgical complications, and elevated mortality in patients with POBL are reported by many other authors.^23–25^ Moreover, biliary leakage is not only associated with increased morbidity and mortality, but also with reduced quality of life.^13,14^

### Biliary Fistula Risk Score

Several analyses have been published in recent years analyzing risk factors for postoperative bile leakage in liver surgery.^16,22,26^

Spetzler et al. reported 14% occurrence of bile leakage in a cohort of 501 liver resections with preoperative chemotherapy, major hepatectomy, and biliodigestive anastomosis as risk factors for postoperative bile leakage.^22^

In our data, BDA was only significant in univariate analysis (38% vs. 12.8%), but not in multivariate analysis.

Ishi et al. identified central hepatectomy and repeated hepatectomy as risk factors for POBL in 310 patients.^27^ Cauchy et al. analyzed laparoscopic major liver resections in 223 patients with POBL 13.5% and found BMI > 28, preceding hepatectomy and biliary reconstruction as independent risk factors.^24^ In our data, mesohepatectomies were not significantly associated with higher bile leakages. Moreover, preceding liver resections did not influence the incidence of POBL. Similar results were reported by Sakamoto and Spetzler.^16,22^

Snyder et al. reported an association with PVE and bile leaks. Those results could not be confirmed in our date, even though PVE was performed for complex operative procedures, e.g., in 17.3% of cases in ALPPS procedures.

Analysis of 297 hemihepatectomies by Zheng et al. with POBL of 30.6% revealed elevated ALAT levels, positive bile culture, BDA, and laparoscopic surgery as independent risk factors for POBL.^25^ Different to that, our data and most studies show significantly lower bile leakage in laparoscopic approaches.^28^

Mohkam et al. established a risk score for severe POBL. In their analyzed cohort ISGLS grade B–C bile leakage were 10.5%. The so-called Mohkam score is based on the identified risk factors: blood loss of > 500 ml, ischemia time > 45 min, anatomical resection, and ALPPS. Noteworthy that all patients receiving BDA were excluded from analysis^21^.

Several of the reported risk scores were tested for our patients. We could not confirm any of the scores. Consequently, we aimed to establish a new score.

In our study, patients’ age, major liver resections, duration of the operative procedure, and combined preceding chemotherapy with FOLFOX and cetuximab were significant risk factors for postoperative bile leakage in multivariate analysis. Nagano et al. described older age as a risk factor for bile leakage, too.^14^ Major resections and longer operating time are frequently identified risk factors for POBL in other studies as well.^16,29^

Different to other studies, only combination of FOLFOX and cetuximab was associated with higher POBL but not single-drug chemotherapy.

Bevacizumab is an angiogenesis inhibitor that might influence the healing process of smaller bile ducts. Guillaud describes bevacizumab as independent risk factor for bile leakage.^23^ Cetuximab has also an anti-angiogenesis effect with similar effects on bile ducts.^30^

Based on those 4 risk factors, the biliary leakage risk score for high (> 15%) and low (< 15%) risk was established with a sensitivity of 67.4% and a specificity of 70.7%.

Our score was evaluated by application in an independent validation cohort of 142 patients.

Validation of the two risk groups showed a specificity of 68.2% and a sensitivity of 75.8%. For the low-risk groups, 7.1% leakages were observed and 34.1% in the high-risk group of the validation cohort.

The scoring system was evaluated retrospectively in the validation cohort. Consequently, no changes in intra- or postoperative management were performed and there is no valid information available regarding the time of detection of POBL within the validation cohort.

Since having established the new scoring system, it was implemented in daily surgical practice in our department. The developed risk score can be easily implemented, e.g., in an Excel sheet, where clinicians can put in the four factors of the individual patient and the risk sore as well as the assignment to a respective risk group is automatically calculated. The BLRS calculator is available in the supplements of this manuscript (Supplement 1).

Currently, we are prospectively evaluating the impact of our new scoring system including each patient receiving liver surgery in our department. The BLRS is calculated for each patient intraoperatively at the end of surgery. Depending on the calculated risk for postoperative bile leakage, decision on abdominal drain placement, biliary duct stenting, etc. is made. Patients with low risk (< 15%) are receiving no abdominal drains, no biliary stenting, and no postoperative antibiotics. Patients with high risk (> 15%) are receiving abdominal drains. In patients with very high probability of biliary leakage (> 50%), prophylactic stenting of the bile duct is performed. Antibiotics are only applied in case of high-risk constellation and preoperative cholestasis or cholangitis. Greater omentum flap is routinely used after major liver resections in our hospital, independently of the BLRS. Fibrin glue is generally not used in liver surgery in our hospital.

Patients with high BLRS are receiving routinely ultrasound on the second to third postoperative day. In case of fluid collection, CT scan is performed and early treatment (antibiotics vs. interventional drain) is initiated.

For prospective evaluation of the BLRS and the initiated measurements, each patient is currently receiving the ultrasound work-up on POD 2–3.

The score is not used for preoperative patient selection so far as the operating time and sometimes the extent of liver resection remain unclear at that time.

However, we are planning to evaluate a “reduced preoperative score” to preoperatively estimate the potential risk for postoperative bile leakage related to age, pretreatment with FOLFOX/cetuximab, and major resection.

### Limitation/Strength of the Current Study

Limitation of our analysis are the retrospective study design as well as the heterogenous patient cohort. Moreover, the exact amount of intraoperative blood loss and performance of white test were not documented. However, the strength of this analysis is that this score is not limited to selected patient cohorts and is true for all malignant diagnoses and any surgical approach (including BDA and ALPPS). Consequently, the BLRS is easily applied in daily practice and can help to estimate patient’s preoperative risk.

## Conclusion

The newly introduced BLRS appears to be a valid marker for the risk of POBL.

Preoperative classification according to the BLRS may aid in patient selection to prevent occurrence of POBL. In the postoperative setting, BLRS may be an indicator for enhanced monitoring, facilitating early detection, and immediate treatment for POBL and may improve postoperative course in liver surgery, prevent adverse events, and thereby decrease overall mortality.

## Supplementary Information

Below is the link to the electronic supplementary material.

Supplementary file1 (XLSX 11 KB)

## Declarations

### Clinical Trial Registration

Due to the retrospective character of the current analysis, clinical trial registration was not performed.
